# Robotic-assisted knee replacement surgery & infection: A historical foundation, systematic review and meta-analysis

**DOI:** 10.1016/j.jor.2023.04.007

**Published:** 2023-04-25

**Authors:** Siddarth Raj, Harroop Bola, Thomas York

**Affiliations:** a University Hospital Coventry and Warwickshire NHS Trust, Coventry, UK; b Imperial College School of Medicine, Imperial College London, London, UK

**Keywords:** Knee, Arthroplasty, infection, Robotic-assisted, Complication, Meta-analysis

## Abstract

**Background:**

An increasing proportion of Knee arthroplasty is performed using robotic-assisted surgical techniques. This study sought to use a meta-analytical approach to establish summary rates of surgical site infection in robotic-assisted procedures and compare the rate of deep infections to those seen in conventional knee arthroplasty.

**Methods:**

This study performed a literature search across four online databases to establish a summary rate of surgical site infection across two categories: deep infection and superficial and pin-site infections. This was processed with the aid of a bespoke data-extraction tool. Risk of Bias analysis was performed using the Cochrane RoB2 tool. Meta-analysis was then performed with tests for heterogeneity and a DerSimonian-Laird random effects model.

**Results:**

A total of 17 studies were identified as appropriate for inclusion in the meta-analysis. The summary rate of overall surgical site infections within one year of robotic knee arthroplasty was found to be 0.568% (SE = 0.183, 95% CI = 0.209–0.927). Deep infections fell to 0.154% (SE = 0.069, 95% CI = 0.018–0.290) and to 0.347% (SE = 0.109, 95% CI = 0.133–0.561) in superficial and pin-site infections.

**Conclusion:**

The surgical site infection rates were found to be low across robotic knee arthroplasty. Further research is required to prove its superiority compared to the conventional, non-robotic technique.

## 1 Introduction

The majority of periprosthetic infections occurring within the first year of surgery are caused by contamination with microorganisms at the time of surgery, either through aerosolised transfer or direct contact with the implant and its surrounding tissues.^1^ In an appropriate host, exceedingly low levels of contamination of the wound can lead to the formation of deep infection.^2^, ^3^, ^4^ Surgical procedures of longer duration result in higher levels of contamination.

Treatment of surgical site infections (SSIs) can be difficult and is associated with poor outcomes and high rates of complications.^5^ Infection is thankfully uncommon, with a primary total knee arthroplasty (TKA) rate of less than 1%.^6^

Robotic-TKA incorporates computerised systems coupled with computed tomography imaging to develop a three-dimensional anatomical reconstruction of the knee joint that is subsequently adopted to guide preoperative and perioperative decisions, including implant positioning and limb alignment.^7^^,^^8^ Robotic-assisted surgery is intended to improve outcomes of TKA by allowing more accurate implant alignment. There is some evidence of the benefit of robotic surgery in terms of length of stay, patient-reported outcomes, and rate of revision, but robotic assistance increases the overall operative duration and involves extra instrumentation of the femur and tibia to insert array pins.^9^^,^^10^ These may theoretically result in an increased risk of infection, but this may be balanced by the purported benefit of less violation of soft tissues when robotic techniques are used.

This systematic review and meta-analysis investigates infection rates following robotic-assisted TKA and unicompartmental knee arthroplasty (UKA). This was confined to those infections occurring within the first year after implantation to reflect the interval over which previous work has shown SSIs to be predominantly determined by peri-operative risk factors.^11^

Further characterisation was targeted by the sub-group of these infections. Specifically, over three categories: deep infection, superficial infection and pin-site infection.

## 2 Methods

### 2.1 Protocol, registration, and search strategy

**Fig. 1:**
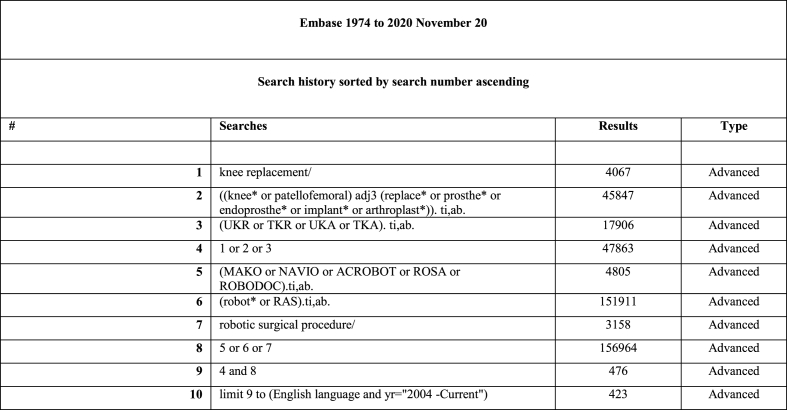
Embase model search strategy.

A review of the Embase, PubMed MEDLINE, Cochrane Database of Systematic Reviews (CDSR), and Cumulative Index to Nursing and Allied Health Literature (CINAHL) databases was performed with a date of publication from 2004 to November 2020 (the date of the search). This period was chosen to reflect the point at which modern surgical robotics became widely available. The search strategy is given in Fig. 1 and contains MESH terms relevant to infection in knee arthroplasty.

### 2.2 Eligibility criteria

Inclusion criteria included studies.

• Written in or translated into English
• Completed during or after the year 2004.
• Original scientific research published in a peer-reviewed academic journal
• The reported intervention of primary robotic/robotic-assisted unicompartmental or total knee arthroplasty with an identified robotic system or systems.
• Recorded the incidence of SSI for the above interventions, with or without further categorisation by the level of infection; deep, superficial, pin-site etc.
• The follow-up period of at least 12 months.
• Identified criteria for patient selection and method of enrolment.

For the purposes of this study, SSI were categorised as infections and inflammatory reactions due to internal knee prosthesis.

### 2.3 Study selection

The search results of each of the databases were combined with the removal of duplicate entries. Titles and abstracts were then screened against the eligibility criteria by the authors, with a decision made for further review or rejection. Those studies warranting further review were further screened by review of their full text. The references of those papers selected for inclusion were individually scrutinised against the same eligibility criteria. Those articles deemed relevant were subsequently included in the review.

### 2.4 Data collection

The following data were collected: the number of patients involved, the number of arthroplasties performed (including categorisation by unicompartmental or total knee arthroplasty), the studied robotic system, and the incidence of SSI (superficial, pin-site, and deep/periprosthetic infection) within the first year. To avoid potentially erroneous assumptions, data on SSIs that was not sufficiently described to categorise further was not included in any sub-group meta-analysis for these values.

### 2.5 Risk of bias assessment

In accordance with the Preferred Reporting Items for Systematic Reviews and Meta-Analyses (PRISMA) guidelines,^12^ the risk of bias was assessed for all studies included in this review. For this, the validated Cochrane Risk of Bias 2 (RoB 2) tool was used in its most recent August 2019 iteration. A cross-sectional assessment was also made to consider the risk of bias across the cumulative evidence of all studies.

### 2.6 Summary measures and synthesis of results

This study's principal summary reporting measures were the overall number of SSIs occurring within one year of the primary operative procedure, sub-classified as deep, superficial or pin site infections. Conventional Q and I^2^ tests for heterogeneity were performed, along with subsequent meta-analysis using a DerSimonian-Laird random effects model in the IBM SPSS 27 software package to generate summary estimates of incidence rates for the principal reporting measures.

## 3 Results

### 3.1 Study selection

**Fig. 2:**
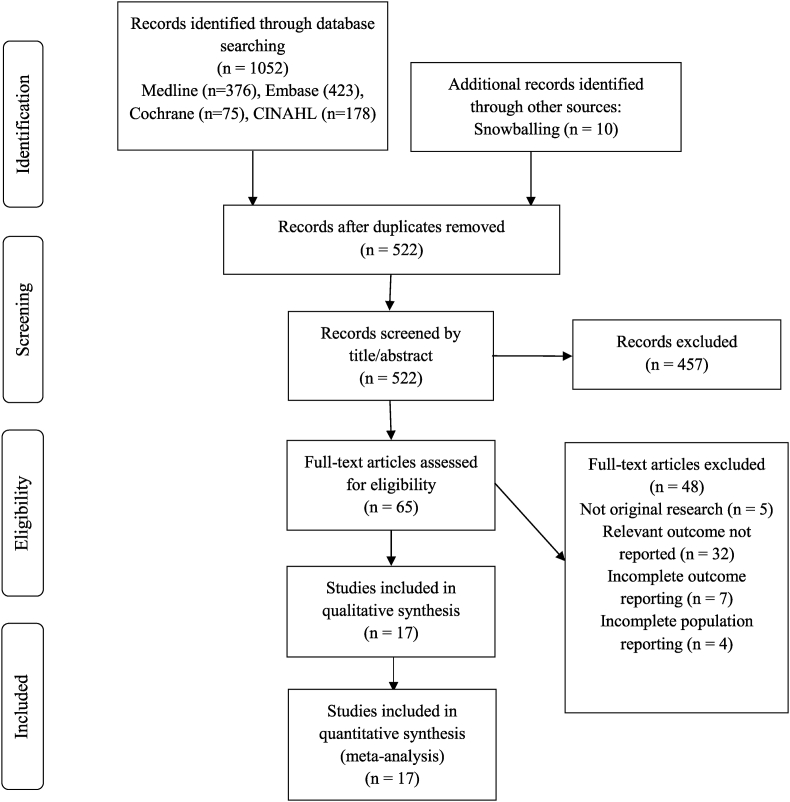
Study selection process.

A total of 1052 studies were identified with the initial search strategy, and a further ten studies were identified through the review of sources. Following study screening and selection, a total of 17 studies were selected.^13^, ^14^, ^15^, ^16^, ^17^, ^18^, ^19^, ^20^, ^21^, ^22^, ^23^, ^24^, ^25^, ^26^, ^27^, ^28^, ^29^ A PRISMA flow diagram is given in Fig. 2.

### 3.2 Study characteristics

The work included in this study reflects a global evidence base, with research from North America, Asia, Europe, and Australasia. These 17 studies referenced a total of 6283 robotic/robotic-assisted knee arthroplasties (unicompartmental n = 4,400, total n = 1883) performed across various surgical platforms.

Duration of follow-up ranged from one to ten years. Individual study characteristics can be seen in Table 1.

**Table 1:** Study characteristics.

Study	Country	Patients Involved	TKA vs UKA	Joint Replacements	Robotic System Used	Surgical Site Infection	Superficial & Pin Site Infections	Deep Infections	Follow Up Period
Battenberg A, 2020^17^	United States	Not Specified	UKA	128	NAVIO	0	0	0	2 years
Dretakis K, 2019^18^	Greece	51	UKA	51	MAKO	0	0	0	3 years
Karakoc, 2018^19^	Turkey	93	TKA	120	MAKO	0	0	0	1 year
Kim K, 2016^20^	South Korea	29	TKA	32	ROBODOC	2	0	2	5 years
Kim Y, 2020^21^	South Korea	850	TKA	975	ROBODOC	17	17	0	10 years
King C, 2020^22^	United States	202	TKA	202	MAKO	0	0	0	1 year
Leelasestaporn C, 2020^23^	Thailand	33	UKA	33	NAVIO (16), MAKO (17)	0	0	0	1 year
Liow M, 2014^24^	Singapore	25	TKA	25	ROBODOC	0	0	0	1year
Liow M, 2017^25^	Singapore	31	TKA	31	ROBODOC	1	0	1	2 years
Lonner JH, 2019^26^	United States	Not Specified	UKA	1064	NAVIO (572), MAKO (492)	5	3	2	1 year
Marchand RC, 2019^27^	United States	335	TKA	335	MAKO	1	1	0	2 years
Marcovigi A, 2017^28^	Italy	Not Specified	UKA	73	MAKO	1	0	1	3 years
Mergenthaler G, 2020^29^	France	175	UKA	200	NAVIO	2	1	1	2 years
Park S, 2007^30^	South Korea	32	TKA	32	ROBODOC	1	1	0	4 years
Song E, 2011^31^	South Korea	30	TKA	60	ROBODOC	0	0	0	1.5 years
St Mart J, 2020^32^	Australia	Not Specified	UKA	2851	MAKO	40	22	18	1.5–2 years
Yang H, 2017^33^	South Korea	71	TKA	71	ROBODOC	2	0	2	10 years
Sum		**>1957**		**6283**		**72**	**45**	**27**	

### 3.3 Risk of bias assessment

**Fig. 3:**
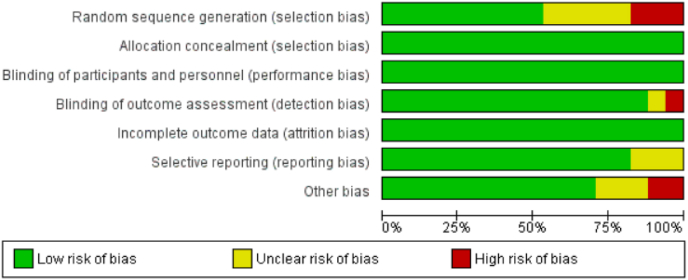
Risk of bias distribution bar chart.

**Fig. 4:**
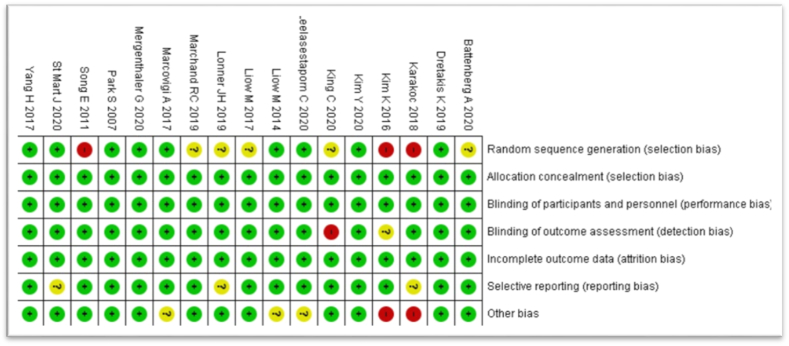
Risk of bias grid.

All included studies were assessed using the Cochrane Risk of Bias (RoB2) tool. Across all papers, there was a judgement of low risk of bias from allocation concealment and the blinding of participants and personnel. Similarly, thorough reporting of the principal summary measures resulted in a globally low assessment of the risk of bias arising from incomplete outcome data (see Fig. 3). In total, only four of the included studies were assessed as having a high risk of bias in any domain. Two of these, Karakoc 2018^15^ and Kim 2016,^16^ were judged to have two domains of high risk and one of unclear risk, respectively; both were subsequently classified as having a moderate overall risk of bias. These were not deemed to be of sufficient concern to warrant exclusion from the meta-analysis. All other studies were assessed as being of low overall concern for bias. Of the total 6283 included procedures, 6131 were drawn from studies with a low risk of bias (see Fig. 4).

### 3.4 Results of individual studies

A total of 72 SSIs were identified, 27 of which were deep infections involving the joint and prosthesis. The remaining 45 were superficial or pin-site infections (Table 2). The highest overall SSI rate was observed in Kim 2016^16^ at 6.25% (95% CI = 0.00–14.91). Seven papers demonstrated a 0% SSI rate; the lowest non-zero rate was in Marchland, 2019^23^^,^ at 0.30% (95% CI = 0.00–0.88). With both two reported SSIs being deep infections, Kim 2016^16^ also displayed the highest deep infection rate (6.25%, 95% CI = 0.00–14.91). Ten papers reported zero deep infections, the lowest non-zero rate of deep infections were shown by St Mart 2020^28^ with 0.63% (95% CI = 0.00–0.92). Regarding superficial and pin site infections, 11 papers reported a 0% rate. The lowest non-zero rate was demonstrated by Marchland 2019^23^ at 0.30% (95% CI = 0.00–0.88). The highest rate of superficial and pin site infections was shown by Park 2017^26^ with 3.13% (95% CI = 0.00–9.25).

**Table 2:** Individual Study results.

Study	Joint Replacements	Surgical Site Infections	SSI Rate (%)	SSI 95% CI	Deep Infections	Deep Infection Rate (%)	DI 95% CI	Superficial & Pin Site Infections	SPSI Rate (%)	SPSI 95% CI
Battenberg A, 2020^17^	128	0	0.000		0	0.000		0	0	
Dretakis K, 2019^18^	51	0	0.000		0	0.000		0	0	
Karakoc, 2018^19^	120	0	0.000		0	0.000		0	0	
Kim K, 2016^20^	32	2	6.250	(0–14.912)	2	6.250	(0–14.912)	0	0	
Kim Y, 2020^21^	975	17	1.744	(0.915–2.572)	0	0.000		17	1.743589744	(0.915–2.572)
King C, 2020^22^	202	0	0.000		0	0.000		0	0	
Leelasestaporn C, 2020^23^	33	0	0.000		0	0.000		0	0	
Liow M, 2014^24^	25	0	0.000		0	0.000		0	0	
Liow M, 2017^25^	31	1	3.226	(0–9.548)	1	3.226	(0–9.548)	0	0	
Lonner JH, 2019^25^	1064	5	0.470	(0.058–0.881)	2	0.188	(0–0.448)	3	0.281954887	(0–0.601)
Marchand RC, 2019^27^	335	1	0.299	(0–0.884)	0	0.000		1	0.298507463	(0–0.884)
Marcovigi A, 2017^28^	73	1	1.370	(0–4.055)	1	1.370	(0–4.055)	0	0	
Mergenthaler G, 2020^29^	200	2	1.000	(0–2.386)	1	0.500	(0–1.480)	1	0.5	(0–1.480)
Park S, 2007^30^	32	1	3.125	(0–9.250)	0	0.000		1	3.125	(0–9.250)
Song E, 2011^31^	60	0	0.000		0	0.000		0	0	
St Mart J, 2020^32^	2851	40	1.403	(0.256–0.585)	18	0.631	(0.340–0.923)	22	0.771659067	(0.450–1.094)
Yang H, 2017^33^	71	2	2.817	(0–6.721)	2	2.817	(0–6.721)	0	0	
Sum	**6283**	**72**			**27**			**45**		

### 3.5 Synthesis of results - overall SSI rate

**Fig. 5:**
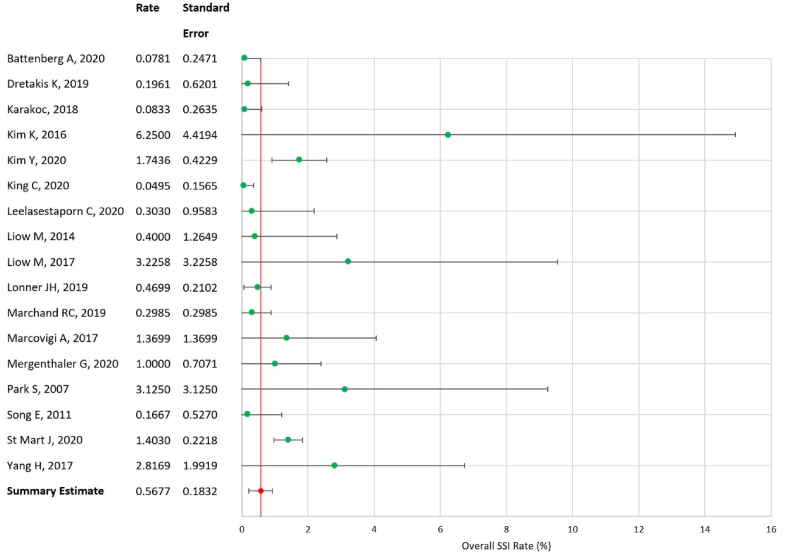
Forest plot of overall SSI rate.

On testing, there was substantial heterogeneity in overall SSI rates amongst studies. Hence a random effects model was used. The summary rate of SSI within one year of operation was found to be 0.568% (SE = 0.183, 95% CI = 0.209–0.927); a forest plot for this data analysis is shown in Fig. 5. No significant variation was identified between the rate of deep infections occurring in total as opposed to unicompartmental robotic knee arthroplasty.

### 3.6 Synthesis of results – deep infection rate

**Fig. 6:**
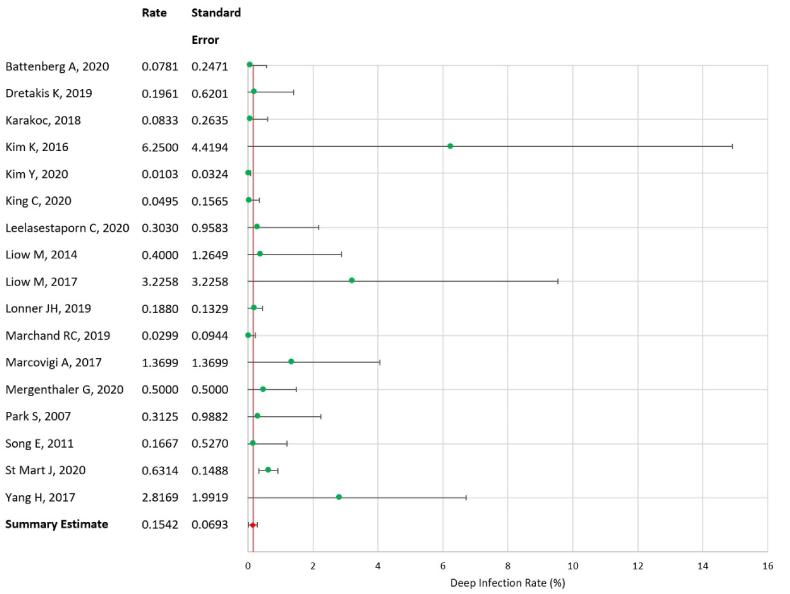
Forest plot of deep infection rate.

Heterogeneity in the rate of deep infections alone was assessed as being lower than that of SSIs overall (Q = 24.84, I^2^ = 35.59). Again, a random effects model was used. The summary rate of deep infection within one year was calculated as 0.154% (SE = 0.069, 95% CI = 0.018–0.290), again with no significant variation in rate between total and unicompartmental arthroplasties. Fig. 6 presents the forest plot of this analysis.

### 3.7 Synthesis of results – superficial and pin-site infection rate

**Fig. 7:**
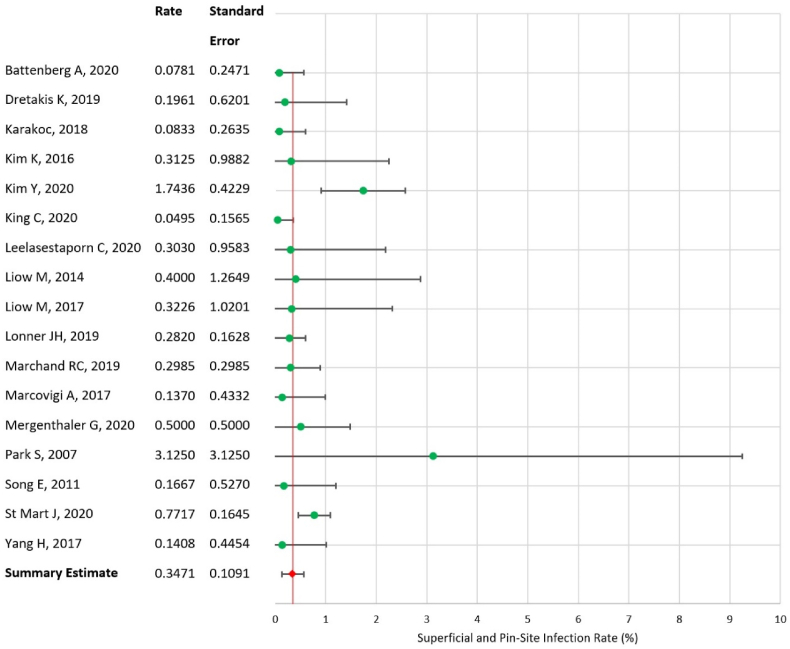
Forest plot of superficial and pin-site infection rate.

Amongst superficial and pin-site infections, the recorded data was found to have a similar degree of heterogeneity to deep infections (Q = 25.04, I^2^ = 36.11). Again, this reflects a moderate degree of heterogeneity and supports the meta-analytic model employed in the study. The summary rate of superficial and pin-site infections within one year of operation was identified as 0.347% (SE = 0.109, 95% CI = 0.133–0.561), again without evidence of variation between total and unicompartmental subtypes. Fig. 7 presents the forest plot of this analysis. Due to insufficiently resolved data in the included studies, it was not possible to fully differentiate pin-site infections from other superficial infections. A discrete analysis of these two sub-groups was subsequently not feasible within this study design.

## 4 Discussion

### 4.1 A low rate of infection

This study identified a low overall rate of SSI in the first year following robotic knee arthroplasty, both for deep and superficial and pin-site infections. These findings are broadly consistent with a wider evidence base indicating that, although serious when they occur, such complications are rarely encountered in clinical practice.^30^

Significant work has been conducted with the aim of eliminating SSIs across arthroplasty,^31^, ^32^, ^33^ including the use of intravenous antibiotics close to the time of skin incision,^6^ antibiotic irrigation and impregnated cement,^34^ clean and laminar airflow, and more novel strategies such as exhaust suits.^35^ These strategies have now been widely synthesised into regional, national,^36^ and transnational^37^ guidelines that are applicable to many of the robotic knee-arthroplasties performed. These peri-operative infection-prophylaxis regimes have been shown to significantly reduce SSIs in conventional knee arthroplasty significantly. Since these same standards of practice apply to robotic knee arthroplasty, it is highly likely that they are exerting an equivalent effect in achieving the low rates of infection observed.

Despite this, a substantial degree of heterogeneity was observed in the overall rate of SSI. The cause for this degree of variation is uncertain but may reflect differences in practice seen across the nine countries with data included in the meta-analysis. It is also possible that advances in robotic knee arthroplasty during the 14-year study period served to reduce the rate of SSIs and therefore introduced heterogeneity. One possible example of this may have been the release of the NAVIO Surgical System in 2016, which can facilitate the use of smaller surgical incisions.^38^ Whilst wider evidence supports the hypothesis that smaller incisions reduce periprosthetic infection rates,^39^ no direct correlation has been reported in robotic knee arthroplasty. The most probable cause for the observed heterogeneity is the limited population size in many of the included studies (see Table 2).

### 4.2 Deep infections

This meta-analysis demonstrated a summary rate of 0.154% regarding deep infection alone. These represent the most clinically significant sub-type of SSIs due to the frequent need for further operative intervention and a significantly associated mortality.^4^^,^^5^^,^^40^

The low summary rate of deep infection supports a consensus that robotic knee replacements represent a safe intervention in appropriately screened patients. This is further supported by the direction of clinical practice, which has seen an annualised growth of approximately 125% in utilising robotic platforms for knee arthroplasty.^41^ Procedural factors associated with robotic surgery may be explanatory of this finding.

Fully active platforms, such as that delivered by the ROBODOC system^20^^,^^42^ allow for a period of operator supervision rather than direct activity during the preparation of the bone surfaces. This may reduce the surgical site's total exposure to human operators, whose proximity has been shown to increase the risk of direct and aerosol contamination of the prosthesis,^43^ an instrumental factor in early peri-prosthetic infection.^1^ However, this is a speculative link and has not yet been shown to affect infection rates in orthopaedic practice. However, evidence suggests a link between greater physical separation of surgeon and site and lower infection rates in robotic laparoscopic procedures.^44^ Further exploration of this hypothesis is warranted.

It has also been shown that the more precise cutting technique employed in robotic knee arthroplasty generates less iatrogenic bone and periarticular soft tissue trauma than conventional procedures.^45^ In particular, there is evidence to suggest that a robotic technique generates lower bone temperatures which do not meet the threshold for thermal necrosis of the bone to occur.^45^^,^^46^ Though largely based on modelling, there is evidence to suggest that thermal necrosis caused by arthroplasty may lead to infective complications.^47^

In contrast to this hypothesis, it should be noted that other research has supposed mechanisms by which robotic surgery may increase the risk of SSI. Foremost among these is increased operative duration, which has been shown to be increased in robotic knee arthroplasty.^40^ Aside from concerns regarding the implications of this on cost per procedure and total surgical efficiency,^48^ evidence has shown that increased mean surgical time is a risk predictor for SSIs in conventional total knee arthroplasty.^49^ This has been presented as an area of concern in several recent publications.^40^^,^^48^ It should be noted, however, that the factors identified as increasing operative time *within* conventional total knee arthroplasty (body mass index, weight, and a total number of comorbidities^49^) are not the causes for an increased operative duration in robotic *as opposed to* convention procedures (learning curve, and theatre setup time^40^).

In general, an interpretation that lower deep infection rates represent a wholesale endorsement of robotic over conventional knee arthroplasty should be avoided. Whilst evidence to support a low rate of deep infections is identified, and though characteristically representing a serious complication, these account for only a small minority of adverse outcomes. Of all indications for revision surgery, the deep infection has been shown to represent between 0.44% and 1.61% of cases.^40^ Consequently, a low rate of deep infection must not be taken as indicative of the wider superiority of robotic knee arthroplasty over conventional methods.

### 4.3 Superficial infections

Individual summary rates of superficial and pin-site infections could not be calculated given the heterogeneity and incomplete classification in the study data. An overall rate of superficial and pin-site infections was identified at 0.347%, equivalent to one in every 288 robotic knee replacements, more than twice the rate of deep infections.

This is consistent with broader findings for SSI rates both in conventional knee replacement^40^ (along with arthroplasty more broadly^50^), which indicate a higher incidence of superficial than deep infections. This is explained by the ready initiation of infection through contamination of the incompletely healed skin and superficial fascial layers with microorganisms native to the patient's skin.^1^ The clinical importance of this mechanism is demonstrated by the predominance of Staphylococcus aureus and Coagulase-negative Staphylococcus species in SSIs associated with knee arthroplasty; these species account for between 46% and 65% of causative organisms.^1^ Whilst the progression of superficial to deep infection is established, the rate at which this occurs in conventional and robotic knee arthroplasty is incompletely understood and represents a meaningful target for future work.

### 4.4 Pin-site infections

Pin site infections are particularly relevant to the operative risks of robotic knee arthroplasty, given that they are induced by a technique not used in conventional procedures. Whilst a very limited number of pin site infections were documented in the included studies,^22^^,^^25^ the generally observed reporting practice was to include them along with other superficial infections. The rationale for this is likely based on their clinical significance; however, a clearer resolution of these findings would represent a valuable means of identifying potential additional risks in robotic knee arthroplasty.

These pin-sites have previously been associated with a small number of other adverse outcomes, including periprosthetic fractures^51^ and neurovascular injury.^9^ Their use cannot be considered benign.

### 4.5 Limitations

Despite a thorough literature search, one significant limitation of this study was the exclusion of data from high-volume joint registry databases, which may have provided a greater degree of power for the meta-analysis. Whilst the potential inclusion of such data was considered early in the design of this study, it was found to be impractical due to the time constraints imposed. The retrieval of information from the UK-based National Joint Registry requires submitting an application to a quarterly review meeting.^52^ this failed to coincide with the study's interval. It should be noted that data from the large volume Australian Joint Replacement Registry was included indirectly through the work of a study identified in the literature search.^28^

As suggested through statistical analysis, there was considerable heterogeneity in the design of the included studies. The risk of bias analysis identified studies conducted on the practice of individual surgeons and insufficient blinding as particular issues. The manner of reporting, particularly regarding differentiating superficial and pin site infections, was also noted as a potential limitation. To control the risk of erroneous inferences being made from this, the two subgroups were combined for the purposes of outcome reporting. The selection of a random rather than fixed effect model was designed to mitigate this when establishing summary rates.

The very low rate of SSIs seen in knee replacement also gave rise to limitations in this study. Primarily, the meta-analysis was found to be statistically underpowered. This issue has been well documented in meta-analytical studies, with research suggesting it to be a limitation in as many as 81% of Cochrane reviews.^53^

Several studies were identified as being individually underpowered,^16^^,^^19^, ^20^, ^21^^,^^26^ all with 50 or fewer joint replacements included in the study. This is observable in the number of studies that reported a zero rate of SSIs. In order to facilitate statistical analysis, a minimal positive constant was applied to these values. Whilst this was a statistically justified approach it will have generated a minor, obligate increase in the summary estimate of all SSI rates calculated in this meta-analysis. Future studies could utilise longer-term follow-up periods, focus on SSI rates exclusively in TKAs or UKAs, and potentially compare SSI rates between different robotic systems.

## 5 Conclusion

This research identified a total of 17 appropriate studies for inclusion in the performed meta-analysis. A DerSimonian-Laird random effects model established a low summary rate of overall SSI in robotic knee arthroplasty (0.568%). This supports an understanding that whilst a potentially catastrophic complication, these infections should not be frequently encountered in routine clinical practice.

Meta-analysis using a random effects model showed that the rate of deep infection, the most deleterious to patient outcomes, was 0.154%. This was significantly lower than the rate of superficial and pin-site infections, which were found to be 0.347%. It was not possible to identify the rate of pin-site infections individually; as these specific complications are only encountered in robotic knee arthroplasty, further study is justified in establishing this.

Hypotheses of increased distance between the surgeon and surgical field in fully active robotic procedures and reduced thermal bone necrosis are identified as potential factors contributing to low infection rates. It is emphasised that this finding should be interpreted cautiously due to limitations arising from the heterogeneity of study data and the low power of the performed meta-analysis.

Whist SSI accounts for only a minority of complications in knee arthroplasty, the high performance of robotic procedures in this regard supports the practice's continued growth. As robotic knee replacement becomes more widespread, an increase in the volume and quality of data relating to infection is expected. This is required to further evidence superiority compared to the conventional, non-robotic technique.

## Ethics approval and consent to participate

Not applicable.

## Consent for publication

Not applicable.

## Availability of data and material

The datasets supporting the conclusions of this review are included within the review and its additional files.

## Competing interests

All authors declare that they have no competing interests.

## Funding

This research did not receive any specific grant from funding agencies in the public, commercial, or not-for-profit sectors.

## Authors’ contributions

**SR** and **HB** performed the search and evaluated titles, abstracts, and then full-text articles to decide on eligible studies to include. For all eligible articles, **SR** and **HB** performed data extraction, including demographics of participants, study characteristics, procedures, and outcomes. Disagreement was resolved via discussion, and where no agreement was reached, an independent third party acted as an arbiter (**TY**). **SR, HB**, and **TY** coordinated and equally contributed to writing the manuscript. **TY** oversaw, reviewed, and edited the manuscript. All authors read and approved the final manuscript.

## Declaration of competing interest

None.
